# Epigenetic Modulation of Vasopressin Expression in Health and Disease

**DOI:** 10.3390/ijms22179415

**Published:** 2021-08-30

**Authors:** Bibiána Török, Csilla Lea Fazekas, Adrienn Szabó, Dóra Zelena

**Affiliations:** 1 Institute of Experimental Medicine, 1083 Budapest, Hungary; torok.bibiana@koki.hu (B.T.); fazekas.csilla@koki.hu (C.L.F.); szabo.adrienn@koki.hu (A.S.); 2 János Szentágothai School of Neurosciences, Semmelweis University, 1085 Budapest, Hungary; 3 Centre for Neuroscience, Szentágothai Research Centre, Institute of Physiology, Medical School, University of Pécs, 7624 Pécs, Hungary

**Keywords:** vasopressin, histone acetylation, DNA methylation, miRNAs

## Abstract

Vasopressin is a ubiquitous molecule playing an important role in a wide range of physiological processes thereby implicated in the pathomechanism of many disorders. Its effect is well characterized through V2 receptors, which regulates the water resorption in kidney, while its vasoconstrictory effect through V1a receptor also received a lot of attention in the maintenance of blood pressure during shock. However, the most striking is its central effect both through the V1b receptors in stress-axis regulation as well as through V1a receptors regulating many aspects of our behavior (e.g., social behavior, learning and memory). Vasopressin has been implicated in the development of depression, due to its connection with chronic stress, as well as schizophrenia because of its involvement in social interactions and memory processes. Epigenetic changes may also play a role in the development of these disorders. The possible mechanism includes DNA methylation, histone modification and/or micro RNAs, and these possible regulations will be in the focus of our present review.

## 1. Vasopressin

Arginine-vasopressin (AVP) is a nonapeptide that is synthesized mainly in the supraoptic (SON), paraventricular (PVN) and suprachiasmatic nucleus of the hypothalamus. In addition, AVP is produced in several other brain areas and organs e.g., medial amygdala [1], bed nucleus of stria terminalis (BNST) [2] or adrenal gland chromaffin cells [3].

The best-known role of AVP, as a hormone, is the regulation of salt-water homeostasis (hence, its other name: antidiuretic hormone), but this special molecule also serves as a neurohormone within the brain involved in more complex mechanisms [4]. Similar to other neuropeptides, AVPergic neurons produce other, classical neurotransmitters (such as glutamate or GABA) as well as other neuropeptides [5]. Indeed, recently, using in situ hybridization, AVP cell groups of hypothalamus and midbrain were found to be glutamatergic, whereas those in regions derived from cerebral nuclei were mainly GABAergic both in rats and mice [6]. In the magnocellular cells of SON, which is the main source of AVP in the blood, colocalization with VGluT2, a marker of glutamatergic cells was found in rats by immunohistochemistry, but the presence of VGluT3 could not be neglected as well [7]. In the PVN, the main center of the hypothalamic-pituitary-adrenal axis (HPA, also known as stress axis), in the parvocellular cells AVP can be found together with corticotropin-releasing hormone (CRH), the main hypothalamic regulator of the axis [8]. Via this way, the AVPergic system participates in the regulation of several physiological processes—beside the above mentioned—from stress hormone release through memory formation [9], thermo- and pain regulation to social behavior [4]. AVP determines behavioral responses to environmental stimuli and participates in the development of social interactions, aggression, reproduction, parental behavior and belonging [4,10,11]. Therefore, alterations in the AVPergic tone may be implicated in the pathology of stress-related disorders (anxiety and depression) [12], Alzheimer’s disorder [13], posttraumatic stress disorder [14] as well as schizophrenia [15,16,17].

AVP exerts its effect through different, V1a, V1b and V2 receptors [18], which have different distributions in the body. The V1a receptor subtype—besides being implicated in vasoconstriction—is the most abundant in the central nervous system; therefore, it has an important role in the regulation of various behavioral and cognitive changes. At the highest concentration, the V2 receptor is found in the kidney; this receptor subtype is responsible for regulating the afore-mentioned salt-water homeostasis. V1b receptors can be found primarily on the anterior lobe of the pituitary; thus, it is involved in the regulation of the stress axis [19].

Taking into consideration the high prevalence of stress and its contribution to practically all kind of diseases, we will later focus on the stress-regulatory aspect of AVP.

### Vasopressin and Stress

**Figure 1:**
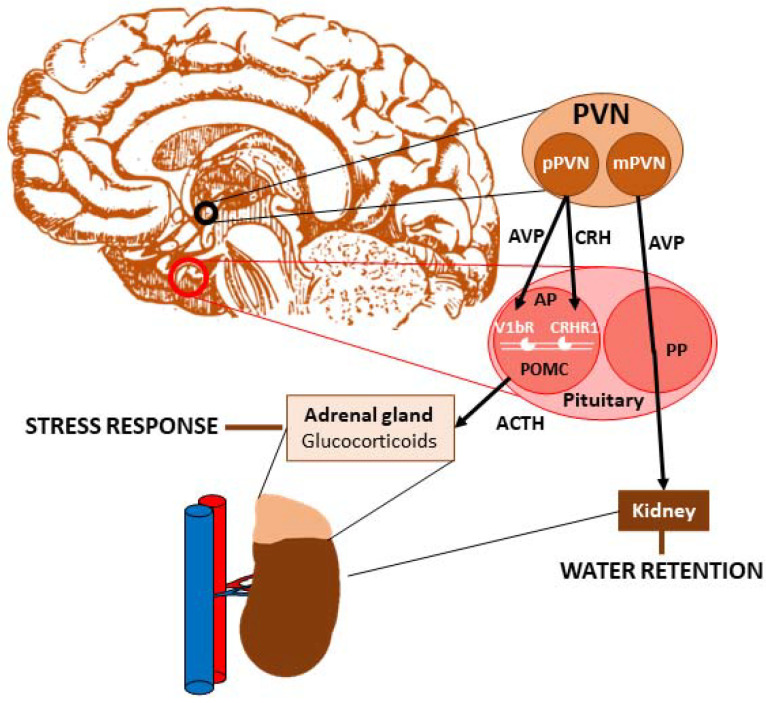
Role of vasopressin (AVP) in stress axis regulation. AVP produced in the magnocellular region of the paraventricular nucleus (PVN) is transported through the posterior pituitary to the blood stream and is responsible for water reabsorption in the kidney. AVP and corticotropin-releasing hormone (CRH) produced in the parvocellular population of PVN are released to the portal circulation of the pituitary, reaching the anterior lobe where they stimulate proopiomelanocortin-producing cells via acting on V1bR and CRHR1 receptors, thereby promoting adrenocorticotropic hormone (ACTH) synthesis and release. In the adrenal cortex, ACTH induces glucocorticoid production (e.g., cortisol), which triggers a response to stress. Abbreviations: ACTH: adrenocorticotropic hormone; AP: anterior pituitary; AVP: vasopressin; CRH: corticotropin-releasing hormone; CRHR1: CRH receptor type 1; mPVN: magnocellular cell population of paraventricular nucleus; POMC: proopiomelanocortin; PP: posterior pituitary; pPVN: parvocellular cell population of paraventricular nucleus; PVN: paraventricular nucleus; V1bR: vasopressin receptor type 1b.

AVP is highly implicated in the regulation of the HPA axis [20] mostly via the above-mentioned V1b receptor subtype [21], however, contribution of the V1a receptors cannot be excluded [22] (Figure 1). In a potentiating manner, together with CRH, AVP stimulates the proopiomelanocortin-producing corticotrope cells of the anterior pituitary to secrete adrenocorticotropin (ACTH). ACTH elevation induces cortisol secretion in adrenal gland (in rodents, the main adrenal gland glucocorticoid is corticosterone); thus, stress response appears.

AVP-induced stress hormone elevation is less sensitive to negative feedback than a CRH-induced one; therefore, it was suggested that this pathway may play a critical role during chronic stress [23]. This theory increased the interest in AVP for developing therapy against stress-related disorder, especially anxiety and depression targeting the V1b receptor. Several preclinical research supported this idea. For example, activation of AVP receptors in the septum of rats has been reported to induce anxiety-related behavior [24]. Moreover, hyper-anxious rats and mice (based upon their performance on elevated plus maze; high anxiety behavior, HAB; low anxiety behavior, LAB) also had higher AVP mRNA expression in their PVN together with disturbances in their HPA axis [25,26]. On the contrary, a Brattleboro rat lacking AVP was less anxious and showed reduced depression-like behavior [12,22] accompanied by changes in HPA axis regulation [27]. However, we should not neglect the V1a receptor contribution. Indeed, in mice, V1a receptor expression was increased in the hippocampus after stress exposure, and these stressors resulted in the development of depressive-like behavior as well [28].

Early studies focused only on V1b receptor, and a highly specific antagonist was developed (SSR149415) [29]. This could be one reason why this compound failed in clinical trials [30]. However, further drug development is still ongoing and recent clinical outcomes with at least two other V1b antagonists (TS-121 and ABT-436) are promising, showing tendencies to reduce the depression scores of patients with major depressive disorder at doses that attenuate HPA axis hyperactivity or block the pituitary V1b receptor [31,32].

Receptors aside, one might target stress-induced epigenetic changes in the AVP gene. Based upon Engel’s biopsychosocial model [33] and its research extension, the three-hit theory [34], the epigenetic programming during early life interacts with the genetic background to shape our overall resilience. Different disorders may be triggered by adult stressors on this background, at our “Achilles heel”, i.e., least resilient organ.

## 2. Early Life Stress and Vasopressin: Possibility for Epigenetic Programming

**Figure 2:**
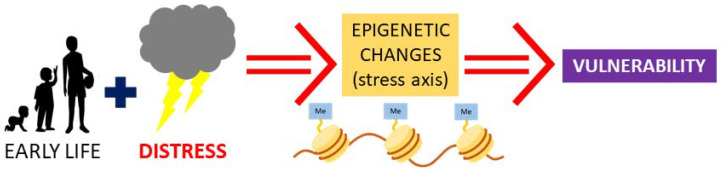
Schematic representation of the effect of early life stress. Epigenetic changes in AVP or AVP receptor genes modifies stress axis function and may also influence the development of vulnerabilityy to various diseases.

Epigenetic mechanisms underlie the molecular background of neural plasticity and neural network formation, a process that requires stable modulation of gene expression [35,36]. Exposure to distress during the critical periods of early life (early life stress; ELS) can cause changes in epigenetic patterns, which modifies development, leading to stable and lasting changes in adulthood [37]. The development of AVP expression and receptor patterns is greatly influenced by the environment, playing a critical role mainly in the fetus and during early childhood. At the same time, AVP regulates the development of the nervous system, and changes in its amount and distribution may have profound behavioral manifestations in adulthood (Figure 2).

In addition to oxytocin, a well-known regulator of labor, lactation as well as social bounding-pregnancy, labor and motherhood induce dynamic changes in the secretion of AVP in mothers and their offspring [38,39,40]. In humans, AVP is detectable in the neurohypophysis at 11–12th gestational weeks, then increases more than 1000-fold in the following weeks [38]. During childbirth, oxytocin-induced uterine contractions stimulate neonatal AVP release [41]. However, this was not based upon a direct measurement, as in humans, the short half-life of AVP makes the equimolarly released co-peptin a better biomarker. Similarly, in rats, AVP synthesis already starts during the prenatal life [42], and receptor expression and distribution patterns gradually change during the first period of life [43]. Accordingly, V1a receptor mRNA and ligand-binding capacity can be detected in rat fetus. However, this undergoes transient pattern changes during the postnatal life and reaches its adult-like distribution around weaning [44]. When and how ELS affects this pattern and later adult life is still under evaluation.

Periodic infant-mother separation during early postnatal life is one of the most commonly used procedures for inducing ELS in rodents. A daily 3 h separation of the pups from their mothers during the first two weeks of life is already enough to induce changes in the hormone homeostasis [45,46]. For instance, it induces a hyperactivity of the HPA axis, resulting in an overshoot of acute stress reaction in adulthood [47,48]. This ESL-induced sustained overactivation of the HPA axis was confirmed to be driven by long-lasting upregulation of AVP expression in PVN parvocellular cells through an epigenetic mechanism [49]. The effect was mediated by the V1b receptor, as its antagonist reversed the molecular and behavioral changes. Several studies have indicated that changes in the AVP content of the hypothalamus may be accompanied by both long-term and short-term behavioral irregularities (see HAB-LAB [25,26] and Brattleboro rats [12,22], for example). There are indications that ELS may alter AVP characteristics in humans as well, and that these may interact with adult predisposition to psychopathology with social dysfunction, such as autism, schizophrenia, depression and personality disorders [50].

All in all, these sparse studies indicate that ELS may program the AVP genes through epigenetic mechanism (presumably changes in DNA methylation), which may lead to changes in the neuroendocrine systems leading to alteration in stress adaptation and behavioral dysfunction.

## 3. Epigenetic Changes in the Vasopressinergic System

Three major mechanisms exist for epigenetic reprogramming. (1) The DNA methylation occurs on cytosine nucleic acids that are followed by guanine in the DNA sequence (i.e., CpG sites) and is rather specifically targeting single genes, mostly resulting in reduced expression. (2) By contrast, changes in chromatin structure may occur via covalent modification of histone and may increase (via histone acetylation) or decrease (via histone methylation) the access of large portions of DNA to the natural subcellular machinery that governs transcription and translation. Unlike histone modification, DNA methylation is a stable signature of the epigenomic status and is therefore most suitable for explaining long-term processes [51]. Therefore, DNA methylation is best studied in relation to AVP and AVP receptor gene changes as well. (3) Besides, there are microRNAs with various functions.

### 3.1. Expression of Genes of the Vasopressinergic System Influenced by DNA Methylation

As introduced above, ELS can cause epigenetic changes both in animal models and humans.

**Figure 3:**
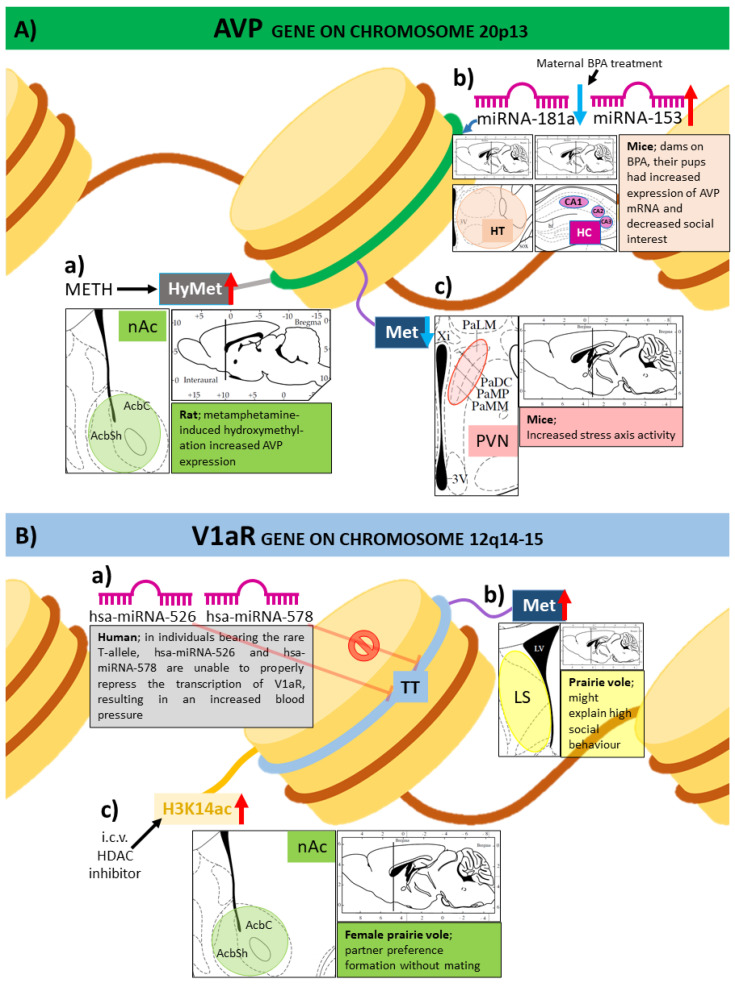
**Increased** vasopressin (AVP, **A**)- or AVP receptor (**B**) gene expression changes linked to epigenetic effects in the literature. (**A**) AVP gene (**a**) Methamphetamine-induced hydroxymethylation (HyMet) increased AVP gene expression in the nucleus accumbens (nAc). AcbC: nAc core region; AcbSh: nAc shell region. (**b**) California mouse dams on diet (before, during and after pregnancy) containing bisphenol A (BPA, commonly found in plastics) gave birth to pups that had increased expression of AVP mRNA in the hypothalamus (HT) and hippocampus (HC), with increased microRNA-153 (miRNA-153) and decreased miRNA-181a levels; CA1-2-3: cornu ammonis 1-2-3 of HC. (**c**) Decreased methylation (Met) was found in mouse nucleus paraventricularis (PVN), which was accompanied by increased AVP gene expression, resulting in stress axis hyperactivity. (**B**) AVP receptor type 1a (V1aR) gene. (**a**) A study about human genome-wide single-nucleotide polymorphisms found an association between the polymorphism of miRNA binding site of the V1aR gene and blood pressure. Two variants were identified: a common G-allele (see in Figure 4). (**B**) and a rare T-allele. In vitro, Homo sapiens (hsa)-miRNA-526 and hsa-miRNA-578 normally reduce V1aR gene expression. However, in the case of T-allele, this repression was either decreased (hsa-miRNA-526) or even completely abolished (hsa-miRNA-578). Individuals bearing homozygous T-allele thus had increased expression of V1aR and higher blood pressure (both systolic and diastolic) compared with homozygous G-allele bearers. TT: thymine-thymine allele variant in V1a receptor gene. (**b**) Hypermethylation on V1aR gene in lateral septum (LS) might contribute to the high social behavior of prairie voles. (**c**) Acetylation increase in Histone 3 Lysine 14 (H3K14ac) by histone deacetylase (HDAC) inhibitors lead to partner preference formation without mating in the nAc of female prairie voles.

Maternal deprivation in mice induced DNA hypomethylation at CpG rich regulatory island (CGI3) region of the AVP gene [49]. As a result, AVP overexpression and high activity of the HPA axis occurred (Figure 3(Ac)). Methyl-binding protein MeCP2 binding sites were methylated at the CGI3 region in parvocellular subdivision of PVN, but not in the magnocellular SON suggesting its contribution to stress adaptation and emotional regulation rather than in salt-water homeostasis.

Parental separation-induced DNA methylation change has also been found in prairie voles [51]. However, these authors were focusing on the lateral septum, an area of the brain that plays an important role in social behavior (Figure 3(Bb)). Increased DNA methylation was detected on septal V1a receptor gene in ELS subjects compared to control. Interestingly, this was accompanied by enhanced rather than the expected reduced V1a receptor mRNA expression. However, it was not easy to make a clear conclusion from this study, as both sex and early parental care influenced the effect of methylation on social behavior. Nevertheless, here, V1a receptor was studied in the septum, which may be influenced by AVP coming from the PVN during stressful conditions [52,53]. Thus, epigenetic changes in AVP system may contribute to maternal separation-induced HPA axis (through V1b receptor) as well as social behavior (via V1a receptor) alterations. However, to explore this in detail, a comprehensive study addressing the PVN and septum simultaneously in a single species would be needed.

**Figure 4:**
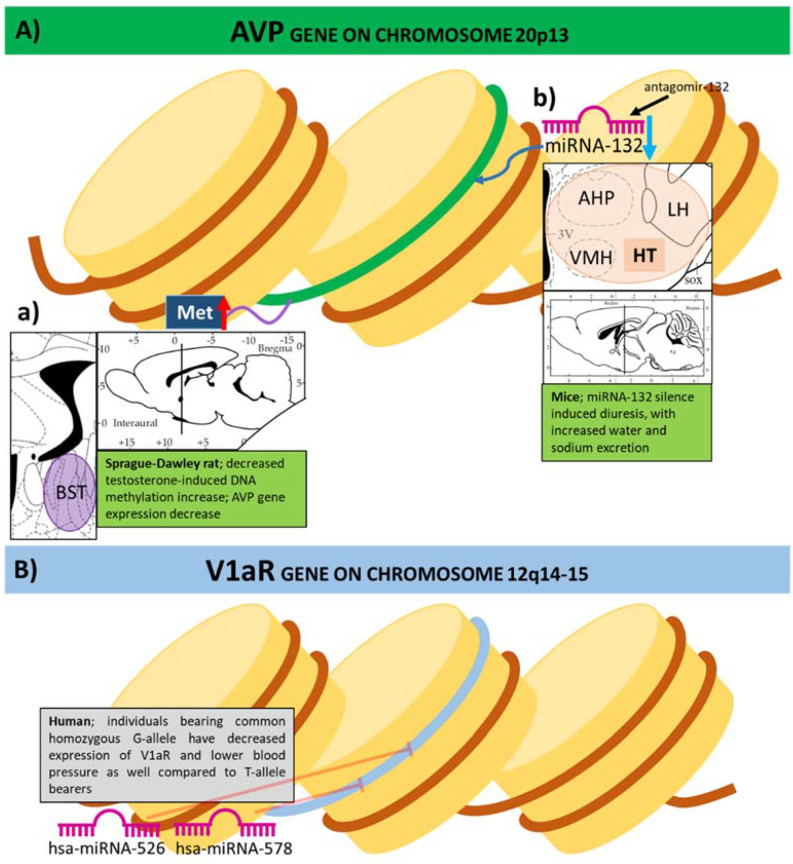
**Decreased** vasopressin (AVP, **A**)- or AVP receptor (**B**) gene expression changes linked to epigenetic effects in the literature. (**A**) AVP gene (**a**) Hypermethylation on AVP gene in bed nuclei of stria terminalis (BST) decreased AVP expression by reduced testosterone levels in Sprague-Dawley rats. (**b**) Antagomir-132-induced microRNA (miRNA)-132 silencing in the mouse hypothalamus (HT) resulted in diuresis with increased water and sodium excretion; AHP: anterior hypothalamus posterior region; LH: lateral hypothalamus; VMH: ventromedial hypothalamus. (**B**) A study about human genome-wide single-nucleotide polymorphisms found an association between the polymorphism of miRNA binding site of the V1aR gene and blood pressure. Two variants were identified: a common G-allele and a rare T-allele (see Figure 3(Ba)). In vitro, Homo sapiens (hsa)-miRNA-526 and hsa-miRNA-578 reduced V1aR gene expression in case of the common G-allele. GT: guanine-thymine allele variant in V1a receptor gene.

Further investigation also showed plasticity of DNA methylation in the brain regulated by testosterone concentration [54]. In male rats, the presence of circulating testosterone helps to maintain the DNA methylation patterns of the AVP promoter regions, especially in the BNST (Figure 4(Aa)). Decreased testosterone levels can cause increased methylation of AVP promoter region leading to reduced AVP mRNA expression. Although epigenetic contribution is not confirmed yet, it is worth mentioning that prolonged (3 month) estradiol treatment in ovariectomized female rats increased AVP mRNA expression in the SON together with changes in social behavior [55]. An epigenetic regulator, methyl-CpG-binding protein 2 (MeCP2), has sex-specific roles during development [56]. When MeCP2 or control siRNA was infused into the amygdala of male rats during the early postnatal period, their AVP mRNA levels went down to the female levels at two weeks and, in adulthood, AVP immunoreactivity and fiber density was also female-like. Thus, a transient reduction in MeCP2 eliminated the sex difference and had lasting impact on AVP expression.

All in all, a special signal (sex hormone) can regulate gene expression pattern in the already developed adult brain most probably through the maintenance of DNA methylation patterns. Direct evidence manipulating the methylation pattern is still missing. Nevertheless, this is a very interesting phenomenon, as it supports the idea that gender differences—also in the AVPergic system [57]—may be underlined by epigenetic changes. In addition, it serves as evidence of the close association that may exist between the steroid hormone system and a nonapeptide with such a wide range of functions as AVP.

Metamphetamine—a psychoactive drug with high addictive potential—can also influence AVP expression by epigenetic changes: it increased TET1- (Ten-eleven translocation methylcytosine dioxygenase 1: an enzyme that might regulate DNA demethylation) and TET3-dependent DNA hydroxymethylation on AVP and CRH genes in rat nucleus accumbens, which is a part of the reward system (Figure 3(Aa)) [58]. Perturbations in the AVPergic pathway might describe the possible mechanism of the stress-relieving effect of the above-mentioned agent, but at the same time might be also responsible for promoting relapses.

A human study showed that acute psychosocial stress exposure dynamically altered DNA methylation of oxytocin (OT) receptor gene measured in the blood cells [59]. Right after stress (10 min), increased DNA methylation was measured compared with pre-stress condition, and shortly thereafter (90 min), it went back to the prestress level. Based upon the structural similarities between AVP and OT (only two amino acids are different), and that they may also act on each other’s receptors, we might assume that there may be a connection between AVP level in pituitary gland and DNA methylation level in OT receptor gene; however, to prove this, further investigation is required. Indeed, in another human study, increased DNA methylation was found on AVP promoter region in connection with alcohol addiction measured by real-time PCR [60]. Nevertheless, these studies suggest that stress-induced plasticity may occur in DNA methylation also in humans.

### 3.2. Histone Modifications and Vasopressin

A few behavioral studies can be found in the literature in connection with AVP and chromatin/histone modification.

Our research group demonstrated the possible role of histone modification in the development of schizophrenia-like behavior in genetically AVP deficient Brattleboro rat (without changes in the enzymes responsible for DNA methylation, DNA methyltransferase, DNMT1 and DNMT3a) [16]. In AVP deficient animals, Western-blot measurements showed lower pan-AcH3 (histone 3 acetylation) immunoreactivity in the frontal part of the brain with elevated levels in the hippocampus. More precise immunohistochemical analysis detected lower H3K9Ac (namely, histone 3 lysine 9 acetylation, H3K9ac) immunopositivity in the prefrontal cortex dorsal peduncular (DP) part and ventrolateral septum. Besides, increased H3K9ac positive cell number in CA1 region of hippocampus was also found. DP H3K9ac immunoreactivity was in negative correlation with prepulse inhibition, which investigates sensorimotor gating deficit (attention deficit can be shown with this test in schizophrenic patients). Furthermore, a positive correlation was found between DP, nucleus accumbens and lateral septum H3K9ac positivity. These regions are elements of the dopaminergic system, and it has been described that dopamine D1 signaling may regulate histone modification [61]. It is also well known that dopamine is highly implicated in schizophrenia [62]. We might assume that reduced AVP signaling in the Brattleboro rats—through histone modification—may influence the dopaminergic system [63,64] leading to development of schizophrenia-like symptoms.

As mentioned above, AVP plays a crucial role in social behavior. It is one of the most important neuropeptides in the regulation of partner preference, biparental care of pups and selective aggression in socially monogamous prairie vole (*Microtus ochrogaster*) [65]. In this species, it has been confirmed that histone deacetylase has a direct effect on pair bonding [66]. I.c.v. injection of histone deacetylase inhibitors has led to histone acetylation at the V1a receptor promoter in nucleus accumbens and caused partner preference formation in females without mating (Figure 3(Bc)). Moreover, after V1a antagonist administration, this effect disappeared.

These preclinical studies suggest a link between histone modifications and phenotypic alteration through changes in AVP or AVP receptor gene expression. However, further studies using histone modifying agent (a hot topic in cancer research [67]) are still required.

### 3.3. MicroRNAs in the Epigenetic Regulation of Vasopressin

MicroRNAs (miRNAs) are short (~22 nt long), non-coding RNA molecules that are responsible for posttranscriptional modification of genes, also known as RNA interference. They are anti-sense and complimentary to the RNA product of target genes and reduce the number of proteins without affecting the number of RNA molecules [68,69,70]. Throughout the years, it has been proven that miRNAs are valuable tools to transiently repress the translation of target proteins in experimental conditions both in vitro and in vivo. However, their exact physiological role and possible usability in therapy is still not clear.

Numerous miRNA molecules are expressed in the kidney, which regulate water excretion and re-uptake. It is not surprising that these miRNAs might operate in accordance with the AVPergic system, as the major role of AVP as a hormone is to promote translocation of aquaporin 2 (AQP2) to the cell membrane in the collecting duct, thereby increasing water resorption. After in vitro ddAVP (synthetic analog of AVP) treatment of the rat inner medullary collecting duct cells, a study using microarray assay found 19 potential miRNA molecules, which responded to the V2 receptor agonist administration with a minimum of 1.3-fold change [71]. Most of them were downregulated, and only one was upregulated, but there were also non-AVP responsive miRNAs. For example, after ddAVP injection, RT-PCR showed the upregulation of miRNA-16, while the product of its target gene, Mylk (myosin light chain kinase), was decreased. At the same time, AQP2 mRNA levels were increased without an effect on the actual AQP2 protein levels.

Further studies focused on the effect of miRNA-132 on AVP system [72]. In a study, antagomir-132 was used to silence miRNA-132 (Figure 4(Ab)). When injected systematically to mice, antagomir-132 induced diuresis, with increased water and sodium excretion, and thus, marked weight loss. This effect was observable up to 48 h after administration. Immunohistochemistry showed that miRNA-132 silencing resulted in re-localization of AQP2 molecules intracellularly from the apical membrane, followed by a marked decrease in protein expression. Moreover, both systematically and centrally injected antagomir-132 decreased the expression of AVP mRNA in the hypothalamus, which could be reversed by the administration of ddAVP. This effect was mediated via one of the miRNA-132 targets, the Mecp2 enhancer region, which can also be found downstream of the Avp gene: while MECP2 gene product was increased, AVP was decreased after silencing miRNA-132 both in vitro and in vivo.

There seems to be clinical relevance of the miRNA-mediated gene silencing in the case of AVP physiology. Even natural compounds, such as the extract of the olive tree leaf, can influence AVP-mediated AQ2 translocation. Namely, the extract inhibited ddAVP induced AQP2 apical membrane trafficking in MCD4 cells via calcium-sensing-receptor (CaSR) activation and Ca^2+^ influx. The effect was confirmed in vivo as well, as rats treated with the extract had an increased mRNA expression of CaSR mRNA and miRNA-137 measured by RT-PCR ([73]; see an excellent book chapter of the molecular mechanisms: [74]).

Interestingly, kidney cells both in immortalized kidney cell lines and in cell cultures from living animals release and take up extracellular vesicles (ECVs) mediated via AVP (ddAVP) and V2 receptor activation. Apparently, this effect was cell selective as the number of ECVs from juxtaglomerular origins did not change in the medium, while it decreased from proximal tubule and cortical collecting duct cell origins. Although the proximal tubule cells were of human origin (HK2 cell lines), and the other two were of mouse origin, the ECV uptake was still induced upon ddAVP incubation. Similar results were obtained when the experiments were repeated in vivo: mice systematically injected with fluorescently labelled ECVs and the V2 receptor antagonist, tolvaptan, had an increase in ECV numbers in the urine. Moreover, the authors confirmed clinical human relevance with a case study of an adolescent patient with diabetes insipidus. After intranasal ddAVP treatment, the ECV amount of glomerular and proximal tubule cell origins increased in the urine samples of this patient. This indicates an evolutionary conservative mechanism, which can be studied both by in vitro and in vivo models [75]. ECVs can contain miRNAs [76,77], and their expression might be modulated in the kidney by AVP-mediated ECV release and uptake. However, more research is needed to understand the exact mechanisms and roles of this phenomenon.

Investigation of miRNA-mediated gene expression in AVP physiology is also relevant during the fetal development and might have life-long lasting effects. Endocrine-disrupting chemicals negatively affect hypothalamic neuroendocrine cell survival during prenatal life [78]. California mouse (*Peromyscus californicus*) dams on diet containing bisphenol A (BPA, commonly found in plastics) before, during and after pregnancy gave birth to pups who had increased expression of AVP mRNA in the hypothalamus and hippocampus (among many other brain areas) (Figure 3(Ab)). At the same time, their miRNA-153 levels were increased and miRNA-181a levels were decreased, suggesting epigenetic contribution to AVP changes. The treatment also decreased social interest toward novel conspecifics and the animals emitted more, but shorter, ultra-sound vocalization, indicating anxiety-like behavior. This is in accordance with the known role of AVP in social behavior [53,65].

Human studies also investigated the role of miRNAs using genome-wide single-nucleotide polymorphisms (SNPs) (Figure 3(Ba) and Figure 4B). An association was found between the polymorphism of miRNA binding site of the V1a receptor gene and blood pressure in Caucasian populations [79]. Two variants were identified: a common G-allele and a rare T-allele. In vitro, hsa-miRNA-526 (hsa: Homo sapiens) and hsa-miRNA-578 reduced V1a receptor gene expression in case of the common G-allele, while, in the case of T-allele, this repression was either decreased (hsa-miRNA-526) or even completely abolished (hsa-miRNA-578). Thus, individuals bearing homozygous T-allele have increased expression of V1a receptor and higher blood pressure (both systolic and diastolic) as well, compared with homozygous G-allele bearers.

In relation to miRNAs, research focused on the classical role of AVP, namely salt-water regulation and blood pressure control through vasoconstriction. However, as an indirect evidence of miRNA-AVP connection during stress, it was shown that ELS—combined with morphine administration—induced an upregulation of AVP mRNA in the hippocampus together with downregulation of several miRNA expression [80].

## 4. Conclusions

An increasing body of evidence confirms epigenetic contribution to changes in AVP or AVP receptor mRNA level, not only during the early perinatal period (e.g., induced by ELS), but also in adulthood. DNA methylation is more targeted on a single gene; therefore, it is better characterized in relation to AVP. However, some hint for bidirectional interaction with histone acetylation was also described. To date, miRNAs are implicated in the hormonal, peripheral role of AVP, and less is known about their interaction regarding behavioral alteration. We have to admit, however, that most of the studies remain at the descriptive level, and confirmation of direct interaction by pharmacological modification of the epigenetic changes is still pending.

## Data Availability

Not applicable.
